# Correlation Between Oral Health and General Medical Condition in Geriatric Patients

**DOI:** 10.3290/j.ohpd.c_2487

**Published:** 2026-03-11

**Authors:** Naomi Sofie Strobel, David Kiramira, Roland Hardt, James Deschner, Philipp C. Mildenberger, Michael Mohr

**Affiliations:** a Naomi Sofie Strobel Dentist, PhD Student, Department of Periodontology and Operative Dentistry, University Medical Center, Johannes Gutenberg University, Mainz, Germany. Study conception and design, conducted the literature review, performed data collection, carried out the statistical analysis, and wrote the manuscript.; b David Kiramira Dentist, Postdoc, Department of Periodontology and Operative Dentistry, University Medical Center, Johannes Gutenberg University, Mainz, Germany. Support in the literature review, assisted in identifying and selecting relevant scientific sources, contributed to the interpretation of dental findings, and critically revised and edited the manuscript for clarity, accuracy, and scientific quality.; c Roland Hardt Professor, Geriatric Department, University Medical Center, Johannes Gutenberg University, Mainz, Germany. Supervised the study, contributed to the study design and clinical interpretation of the results, and critically revised the manuscript for important intellectual content.; d James Deschner Professor, Department of Periodontology and Operative Dentistry, University Medical Center, Johannes Gutenberg University, Mainz, Germany. Supervised the dental aspects of the study, contributed to the study design and interpretation of dental findings, and critically revised the manuscript; e Philipp C. Mildenberger Statistician, Institute of Medical Biostatistics, Epidemiology and Informatics, University Medical Center, Mainz, Germany. Performed and supervised the statistical analyses and contributed to the interpretation of the statistical results.; f Michael Mohr Physician, Postdoc, Geriatric Department, University Medical Center, Johannes Gutenberg University, Mainz, Germany. Informed and recruited the patients, contributed to the clinical and geriatric aspects of the study, assisted in the collection of relevant literature sources, and critically revised the manuscript.

**Keywords:** Barthel index, DMFT index, geriatric, oral health, Tinetti mobility test.

## Abstract

**Purpose:**

The aging population presents an increasing demand for healthcare, including dental care. Oral health in older adults is often compromised by physical limitations, chronic diseases, and medications. This study investigates the oral health status of geriatric patients and its correlation with general medical, psychosocial, and functional health indicators.

**Materials and Methods:**

A cross-sectional observational study was conducted from January 2022 to September 2023, involving 62 hospitalized patients (aged 69–99) in a geriatric unit. Dental health was assessed using the DMFT index, and geriatric assessments included the Barthel Index, Tinetti mobility test, and hand strength measurements. Participants’ demographic and clinical data, such as marital status, living situation, and care level, were recorded. Descriptive and inferential statistical methods were used for data analysis.

**Results:**

A statistically significant association was observed between the level of geriatric impairment and oral health status, as assessed by the DMFT index, in older patients. Cognitive function, measured by the Mini-Mental State Examination (MMSE), showed a strong relationship with oral health. Participants with severe dementia had a statistically significantly higher DMFT index (p < 0.001) and a greater number of missing teeth (p < 0.001) compared to those without cognitive impairment. Mild cognitive impairment was associated with an increased number of decayed teeth (p = 0.044), while severe dementia was linked to a statistically significant rise in the number of decayed teeth (p < 0.001). Physical function, as assessed by the Barthel Index, was also statistically significantly associated with oral health. The DMFT index was higher among participants with mild (p = 0.007) and severe dependency (p = 0.033) compared to independent individuals. The number of missing teeth was statistically significantly greater in participants with mild (p < 0.001), moderate (p = 0.049), and severe dependency (p = 0.013). Furthermore, those who were totally dependent had a statistically significantly higher number of decayed teeth (p = 0.039).

**Conclusion:**

Oral health is crucial to overall health and quality of life in the elderly. Integrating dental assessments into geriatric care is essential, and tailored dental treatment strategies are needed to improve the well-being of geriatric patients. Future research should explore causal relationships between oral health and geriatric diseases to refine care strategies.

Due to an aging society, the oral health of geriatric patients in particular is an increasingly important topic in dentistry. While in 1950 the proportion of over 69-year-olds was only 7%, it was 18% in 2025, and is expected to reach 25% in Germany by 2070.^7^ Consequently, it is anticipated that dental practices and clinics will experience an influx of geriatric patients. This raises several critical questions: How can geriatric patients be characterized from a dental perspective, taking into account the unique challenges and specific needs they present? What common complaints and dental issues are encountered in this population, and how do these impact the delivery of care in dental practice? It is also necessary to examine whether there are significant differences in dental findings and treatment strategies for geriatric patients compared to the general patient population. Additionally, the treatment of geriatric patients presents particular challenges, which require a deeper understanding of the best approaches to overcome them. A key consideration is how to effectively engage and involve geriatric patients in dental care, especially since some may face difficulties in making or attending appointments or may not perceive the urgency of dental visits relative to other medical consultations. Financial barriers such as limited income, fixed pensions, high out-of-pocket expenses, and insufficient insurance coverage can significantly hinder access to and continuity of dental care. Additionally, the need for comprehensive medical and nursing care in geriatric patients with poor general health places a substantial financial burden on healthcare systems and, in some countries, on social welfare structures. A thorough understanding of these socioeconomic factors is essential for developing targeted strategies that address the specific needs of this population and support the provision of appropriate and sustainable oral healthcare.

The fifth German Oral Health Study from 2016 revealed that dental diseases are shifting to old age as a result of demographic developments, and mainly to people in need of nursing care.^11^ Overall, there were fewer edentulous senior citizens and, on average, with five more of their own teeth than in 1997. The large-scale socio-epidemiological study also shows more fixed dentures among younger senior citizens (65- to 74-year-olds). In contrast, however, removable dentures are more common, as is a higher caries experience among senior citizens with nursing care needs; almost 30% of seniors with nursing care needs are no longer able to clean their teeth and dentures themselves. Moreover, 60% of senior citizens with nursing care needs are no longer able to organize a dentist appointment and then visit the practice. This study therefore emphasises the new challenges for dental therapy and nursing-care concepts due to the shift of dental diseases to old age, especially in dealing with patients in need of long-term nursing care.^11^

In everyday life, the mobility of older people is often limited, which may be one of the reasons why it is increasingly common for dentists not to carry out regular dental check-ups.^13^ If continuous check-ups are not carried out, pathologies cannot be recognized and treated at an early stage. In addition to the frequently occurring physiological and age-related diseases such as dry mouth, this can be a reason for multimorbidity in the dentition of the elderly patient group. It is now known and proven that untreated caries and tooth loss are prevalent on a global level with wide variations among different countries, age groups, and socioeconomic statuses.^2^

The average geriatric patient is over 80 years old. A holistic approach is necessary for their treatment, as many senior citizens suffer from several illnesses at the same time, some of which are chronic. Specific symptoms cannot be treated in a targeted manner.^9^ A connection between the well-documented general medical multimorbidity of geriatric patients and their oral health is likely. Various geriatric assessments can describe well the health and general condition of geriatric patients, such as the Barthel Index, which describes the degree of a patient’s independence in activities of daily living.^18^ Evidence shows that the frequency of falls increases with age.^5^ The Tinetti mobility test is a reliable instrument for evaluating the risk of falling.^23^ Studies have shown that measuring hand strength with a dynamometer can provide information on patients’ oral hygiene. A study from Korea confirms that a correlation between higher DMFT index values and lower measured hand strength was demonstrable.^15^

The link between oral health and systemic medical conditions has been increasingly substantiated in recent literature, particularly in relation to periodontal diseases. Studies such as that of Chalmers and Hernandez-Kapila^3^ have highlighted the role of the oral microbiome and periodontal inflammation in the pathogenesis of neurodegenerative diseases like Alzheimer’s. Eke et al^6^ and Van der Velden^24^ have demonstrated both the predictive potential and long-term progression of periodontal disease using clinical and self-reporting tools. On a broader scale, research by Isola et al^12^ and Villoria et al^25^ underscores the bidirectional relationship between periodontal conditions and systemic diseases, confirming periodontitis as a systemic health concern. Furthermore, the work of Sanz et al^21^ illustrates the growing international recognition of periodontology’s role in general health.

Although the systemic effects of periodontal disease are well established, less focus has been placed on more readily accessible indicators of oral health, such as the DMFT (Decayed, Missing, and Filled Teeth) index, and their potential relationship with general health, particularly in geriatric populations. This study aims to investigate these associations, emphasizing the DMFT index as a simple and non-invasive measure of oral health. The objective is to equip geriatricians and other non-dental healthcare providers, including nursing staff, with a practical tool for rapid oral health assessment. Ultimately, this approach may enhance a more comprehensive understanding of the patient’s overall health and support the implementation of integrated, patient-centered care strategies.

Our study investigates the oral health of geriatric in-patients. Its purpose is to describe the dental condition of geriatric patients in order to emphasize the need for treatment and to contribute to new nursing care concepts with valuable results. The additional oral findings represent an extension of the overall geriatric findings and are intended to help patients be viewed holistically and ultimately treated accordingly. Specifically, the aims are to A) investigate whether there are differences in the oral findings based on demographic and clinical factors, including gender, marital status, and nursing care dependency; B) investigate potential correlations between general health indicators, geriatric assessments, and dental findings; and finally C) propose integrated nursing care strategies to improve the oral and overall health of elderly patients. This study hypothesizes that there is a statistically significant association between the degree of geriatric impairment in older patients as measured by standardized assessments and oral health status as quantified by the DMFT index.

## MATERIALS AND METHODS

### Patients and Study Design

This cross-sectional observational study was carried out at the Geriatric Department of the Mainz University Medical Center between January 2022 and September 2023. The Declaration of Helsinki and the recommendations of Good Clinical Practice (GCP) and Good Epidemiological Practice (GEP) were strictly followed throughout the study. Approval was obtained from the Ethics Committee of the State Medical Association of Rhineland-Palatinate (Landesärztekammer Rheinland-Pfalz, Application No. 2022-16574).

Participation in the study was voluntary. Prior to participation, oral and written information about the study procedure was provided. Written informed consent was obtained from the patient. In the case of patients who were not capable of giving consent, informed consent for was provided by the legal guardian. No disadvantages arose for patients regardless of participation/non-participation.

A total of 62 geriatric patients were examined. Most of them had already been hospitalized on the geriatric ward for 1–2 weeks, while others were examined during the first few days of their stay. Beforehand, they were informed by a geriatrician about the study and the procedure.

### Oral and Geriatric Assessments

After general and special anamnesis as well as various geriatric assessments were taken, a dental anamnesis and detailed oral status were recorded. The general medical history included age, gender, BMI, marital status, living situation, level of nursing care, and diet.

In Germany, there are five levels of nursing care that make it possible to describe the type and severity of physical, mental and/or psychological impairments of patients in need of care. The nursing care level also determines which subsidies insured persons receive from their nursing care insurance. The level of nursing care is based on the severity of the impairment of the independence or abilities of the person in need of nursing care and is graded from minor impairments of independence or abilities (level of nursing care: 1) to the most severe impairments of independence or abilities that are associated with special requirements for nursing care (level of nursing care: 5). The level of care nursing is determined by an assessor. The assessor uses six modules to determine how much help a person needs to cope with everyday life. Each module contains up to 16 different criteria, which are assessed using a points system.^22^

Geriatric assessments of interest included:

Mini-Mental State Examination (MMSE): A practical method for grading the cognitive state of patients. It assesses domains such as orientation, attention, memory, language, and visuospatial skills. The test consists of 30 points, with higher scores indicating better cognitive performance.^8^
Barthel index: Assesses a person’s ability to perform ten basic activities of daily living, including eating, bathing, grooming, dressing, bowel and bladder control, toileting, transferring (e.g., from bed to chair), mobility, and climbing stairs. Each activity is scored on a scale of 0 to 10, with higher scores indicating greater independence and functional ability. A maximum of 100 points can be achieved.^18^
Hand strength measurement: A performance test to determine hand strength, which is said to have a positive correlation with overall body strength and a negative correlation with the risk of falls and fractures, as well as overall mortality. It is typically assessed using a dynamometer, with the patient exerting maximum force with the dominant hand.^20^
Tinetti mobility test: A performance test to examine balance and gait, primarily to evaluate fall risk. The test consists of two components: a balance section (maximum 16 points) and a gait section (maximum 12 points), totaling up to maximum of 28 points possible.^14^

Dental assessments included dental anamnesis, detailed oral examinations, and calculation of the DMFT index. The additional dental anamnesis asked about the patient’s dental care; whether they still had their own teeth or dentures, and if so, which ones; whether they still brushed their own teeth, with what, and how often per day. In addition, questions were asked about subjective dry mouth, chewing ability, and pain. In addition to the general findings, a detailed oral status survey also included periodontological findings, such as probing depth, gingival margin, bleeding on probing, furcation, plaque, and mobility.^10^ However, the periodontal findings collected during the study will be evaluated in a separate analysis. This publication specifically concentrated on the relationship between geriatric and general medical parameters and oral health as measured by the DMFT index in the geriatric population.

The DMFT index represents the total number of decayed, missing, and filled teeth and ranges from 0 to 28, or from 0 to 32 if wisdom teeth are included. A DMFT index of 0 therefore describes a completely dentate patient with only healthy, unaltered teeth.^16,26^ This study included the wisdom teeth in the DMFT index for greater accuracy.

### Statistical Analysis 

The statistical analyses were carried out using SPSS (version 23 V5 R, IBM; Armonk, NY, USA). The descriptive data were visualized as tables. Finally, univariable Poisson regression with robust variance estimation was used to determine the relationship between each potential influencing factor and the examined oral health as measured by the DMFT index or number of healthy teeth, as well as the individual number of decayed, missing, and filled teeth.

## RESULTS

### Characterization of the Study Population

A total of 62 geriatric patients (41 females and 21 males) aged between 69 and 99 years were examined. The mean age of the patients was 83.81 years (± 6.17). The study population included 31 widowed patients, 19 married patients, 11 single patients, and 1 patient who did not wish to state their marital status. Thirty-nine out of 62 patients stated that they lived alone, the remaining 23 alone with help. In terms of nursing care level of the study population, 29 patients had a nursing care level 0, 6 had nursing care level 1, 15 had nursing care level 2, 11 had nursing care level 3, and 1 patient had nursing care level 4. The mean BMI was 25.64 (± 5.23). In terms of diet, 55 of 62 patients stated that they ate whole foods, 6 ate soft food, and 1 ate mashed food (Table 1).

**Table 1:** Characteristics of the study population, including social and medical aspects

Variables		n (%)	Mean ± SD
Sex	Female	41 (66.1)	
	Male	21 (33.9)	
Age	All study patients	62 (100)	83.81 ± 6.17
	Female	41 (66.1)	84.05 ± 0.94
	Male	21 (33.9)	83.33 ± 1.43
Marital status	Single	11 (17.7)	
	Married	19 (30.6)	
	Widowed	31 (50.0)	
	Missing information	1 (1.6)	
Living environment	Alone	39 (62.9)	
	Alone with help	23 (37.1)	
Level of care	0 (no impairment of independence)	29 (46.8)	
	1 (minor impairment of independence)	6 (9.7)	
	2 (significant impairment of independence)	15 (24.2)	
	3 (severe impairment of independence)	11 (17.7)	
	≥4 (severe impairment of independence with special requirements for nursing care)	1 (1.6)	
Body Mass Index (BMI)	Underweight (BMI<18.5)	3 (4.8)	25.64 ± 5.23
	Normal weight (18.5≤ BMI<25)	26 (41.9)	
	Overweight (25≤BMI<30)	24 (38.7)	
	Obese (BMI≥30)	9 (14.5)	
Diet	Pureed diet	1 (1.6)	
	Soft diet	6 (9.7)	
	Wholefood diet	55 (88.7)	


### Oral Parameters 

Twenty-two patients still had only their natural teeth, 26 had their natural teeth in combination with a denture, and 14 had solely dentures.

Concerning daily oral hygiene, 59 out of 62 patients stated that they still brushed their teeth alone, while the remaining 3 needed help. Of these, 39 patients stated that they used a manual toothbrush, 4 used a manual toothbrush and additional devices for interdental care such as dental floss or interdental brushes, 11 an electric toothbrush, and the remaining 8 used an electric toothbrush and additional devices for interdental care. The frequency of dental hygiene practice per day was stated by 13 patients as once, by 28 as twice, and by 18 as three times per day. Three patients did not practice oral hygiene at all.

Fifty-four out of 62 patients stated that they were still able to chew well, 43 out of 62 reported subjective dry mouth, and only one out of 62 patients stated that they were currently in pain. There were no patients with a DMFT of 0; the mean DMFT index was 27.06 (± 6.20) with 1.11 (± 3.06) decayed, 19.63 (± 9.45) missing, and 1.15 (± 1.96) filled teeth, or rather 4.94 (± 6.20) completely healthy teeth.

The average patient in this study had 12.37 (± 9.44) teeth, 5.42 (± 4.60) in the maxilla and 6.95 (± 5.25) in the mandible, or rather 4.15 (± 3.15) front teeth, 2.56 (± 1.57) canines, 3.13 (± 2.74) premolars, and 2.13 (± 2.56) molars. In addition, there was a mean of 1.53 (± 2.26) occluding pairs. Overall, no patients had 32 healthy teeth. With a mean of only 2.13 (± 2.56) out of 12 possible molars and 3.13 (± 2.74) out of 8 possible premolars, this population was well below the percentage of anterior teeth (mean 51.88%) and canines (mean 64%), at 17.75% and 39.13%, respectively. This is particularly significant with regard to the mouth-related quality of life due to possibly limited food comminution due to the lack of occluding pairs (Table 2).

**Table 2:** Characteristics of the study population, geriatric assessments

Variables		n (%)	Mean ± SD
Mini-Mental State Examination (MMSE)	No impairment (30–27 points)	33 (53.2)	25.39 ± 3.83
	Mild cognitive impairment (26–21 points)	24 (28.7)	
	Moderate dementia (20–10 points)	4 (6.5)	
	Severe dementia (<10 points)	1 (1.6)	
Barthel index	Independent (100–90 points)	3 (4.8)	50.00 ± 18.67
	Mild dependency (89–75 points)	3 (4.8)	
	Moderate dependency (74–50 points)	26 (41.9)	
	Severe dependency (49–25 points)	27 (43.5)	
	Total dependency (<25 points)	3 (4.8)	
Hand grip	Normal strength (≥25 kg)	17 (27.4)	15.63 ± 7.49
	Weak grip strength, risk of sarcopenia (<25 kg)	45 (72.6)	
Tinetti index	Low fall risk (≥24 points)	8 (12.9)	12.98 ± 7.05
	Moderate fall risk (19–23 points)	5 (8.1)	
	High fall risk (≤18 points)	49 (79.0)	


### Geriatric Parameters

The mean MMSE score was 25.39 (± 3.83), which indicates mild cognitive impairment (MCI), as it falls slightly below the normal cognitive range (26–30 points).

The mean Barthel index was 50.00 (± 18.67), which indicates moderate dependence in activities of daily living. Patients with this score require considerable assistance with basic self-care tasks but may retain some degree of independence in certain activities. This level of functional impairment suggests the need for ongoing support.

The mean hand-grip index was 15.63 (± 7.49), indicating reduced muscle strength, which may be associated with sarcopenia, frailty, or general physical decline, particularly in geriatric patients.

Finally, the mean Tinetti index was 12.98 (± 7.05) in our study population. A mean Tinetti Index score of 12.98 indicates a moderate risk of falls, reflecting impaired balance and gait (Table 3).

**Table 3:** Characteristics of the study population, oral aspects

Variables			Mean ± SD	
Number of teeth	Maxilla		5.42 ± 4.60	12.37 ± 9.44
	Mandible		6.95 ± 5.25	
	Incisors		4.15 ± 3.15	
	Canines		2.56 ± 1.57	
	Premolars		3.13 ± 2.74	
	Molars		2.13 ± 2.56	
	Occluding pairs		1.53 ± 2.26	
	Healthy, non-decayed, non-filled		4.94 ± 6.20	
	Decayed		1.11 ± 3.06	
	Missing		19.63 ± 9.45	
	Filled		1.15 ± 1.96	
DMFT index			27.06 ± 6.20	
Dental prosthesis status			n (%)	
	Natural teeth		22 (35.5)	
	Natural teeth + dentures		26 (41.9)	
	Dentures		14 (22.6)	
Ability to chew	Yes		54 (87.1)	
	No		8 (12.9)	
Dry mouth	Yes		43 (69.4)	
	No		19 (30.6)	
Pain	Yes		1 (1.6)	
	No		61 (98.4)	
Oral hygiene practice	Dependency	Independent	59 (95.2)	
		Dependent	3 (4.8)	
	Frequency	0/d	3 (4.8)	
		1/d	13 (21.0)	
		2/d	28 (45.2)	
		3/d	18 (29.0)	
	Devices	Manual toothbrush	39 (62.9)	
		Manual toothbrush + interdental care	4 (6.5)	
		Electric toothbrush	11 (17.7)	
		Electric toothbrush + interdental care	8 (12.9)	


#### Correlation between oral and geriatric parameters

The reported findings summarize the statistically significant results of the Poisson regression analyses. Other associations examined in the analysis were not statistically significant, but can be found in the tables.

#### Geriatric parameters correlated with healthy teeth (non-caries and non-filled) 

When correlating the number of healthy, non-decayed, non-filled teeth with the patient’s gender, it was found that women had 61% fewer healthy teeth compared to men, which was statistically significant. Patients with a nursing care level of 1 had 89% fewer healthy teeth compared to patients with a nursing care level of 0, patients with a nursing care level of 2 had 65% fewer healthy teeth compared to patients with a nursing care level of 0, and patients with a care level of 3 had 46% fewer healthy teeth compared to patients with a nursing care level of 0 (Table 4).

**Table 4:** Poisson regression, variable, number of healthy, non-decayed, non-filled teeth

Correlated variable		Number of healthy teeth (mean ± SD)	Comparison		Relative risk	p-value
Correlated variable		Number of healthy teeth (mean ± SD)	Comparison		Relative risk	p-value
Sex	Male	8.29 ± 1.72				
	Female	3.22 ± 0.67	Female vs male		0.39	0.001**
Marital status	Married	7.32 ± 1.63	Married vs single		2.51	0.026*
	Single	2.91 ± 1.07				
	Widowed	4.35 ± 1.10	Widowed vs single		1.49	0.349
Living environment	Alone	4.23 ± 0.91				
	Alone with help	6.41 ± 1.49	Alone with help vs alone		1.45	0.238
Level of care	0	7.38 ± 1.41				
	1	0.83 ± 0.83	1 vs 0		0.11	0.019*
	2	2.79 ± 0.83	2 vs 0		0.35	0.003**
	3	4.36 ± 1.32	3 vs 0		0.54	0.085
	≥4	0.00 ± 0.00	≥4 vs 0		-	-
BMI	Underweight	10.67 ± 8.74				
	Normal weight	2.96 ± 0.83	Normal weight vs underweight		0.28	0.077
	Overweight	7.42 ± 1.31	Overweight vs underweight		0.69	0.599
	Obese	2.11 ± 0.94	Obesity vs underweight		0.19	0.040*
MMSE	No impairment	5.24 ± 1.13				
	Mild cognitive impairment	5.04 ± 1.29	Mild cognitive impairment vs no impairment		0.96	0.905
	Moderate dementia	3.00 ± 1.78	Moderate dementia vs no impairment		0.57	0.315
	Severe dementia	0.00 ± 0.00	Severe dementia vs no impairment		–	–
Barthel index	Independent	1.67 ± 1.66				
	Mild dependency	7.33 ± 1.86	Mild dependency vs independent		4.40	0.079
	Moderate dependency	4.88 ± 1.42	Moderate dependency vs independent		2.93	0.214
	Severe dependency	5.37 ± 1.13	Severe dependency vs independent		3.22	0.165
	Total dependency	2.33 ± 2.33	Total dependency vs independent		1.39	0.771
Hand grip	Normal strength	6.41 ± 1.79				
	Weak grip strength, risk of sarcopenia	4.38 ± 0.85	Weak grip strength vs normal strength		0.68	0.251
Tinetti index	Low fall risk					
	Moderate fall risk		Moderate fall risk vs low fall risk		0.92	0.896
	High fall risk		High fall risk vs low fall risk		0.48	0.124
* p < 0.05; ** p < 0.01; *** p < 0.001.						

#### Geriatric parameters correlated with DMFT index

When correlating the DMFT index with the patient’s gender, it was found that women had a statistically significant 21% higher DMFT index compared to men. Married people had a 15% lower DMFT index compared to single people, while widowed people had a 5% lower DMFT index compared to single people. Patients with a nursing care level of 1 had a 27% higher DMFT index compared to patients with a care level of 0, patients with a care level of 2 had a 19% higher DMFT index compared to patients with a care level of 0, patients with a care level of 3 had a 12% higher DMFT index compared to patients with a care level of 0, and patients with a care level of 4 had a 29% higher DMFT index compared to patients with a care level of 0. If the geriatric assessment MMSE increases by one unit, the DMFT index decreases by 1% (Table 5).

**Table 5:** Poisson regression, variable, and DMFT index

Correlated variable		DMFT (Mean ± SD)	Comparison	relative risk	p-value
Sex	Male	23.71 ± 1.72			
	Female	28.70 ± 0.68	Female vs male	1.21	0.009*
Marital status	Single	29.09 ± 1.07			
	Married	24.68 ± 1.63	Married vs single	0.85	0.025*
	Widowed	27.65 ± 1.10	Widowed vs single	0.95	0.333
Living environment	Alone	27.77 ± 0.91	Alone with help vs alone	0.93	0.265
	Alone with help	25.59 ± 1.49			
Level of care	0	24.62 ± 1.41			
	1	31.17 ± 0.83	1 vs 0	1.27	<0.001***
	2	29.21 ± 0.83	2 vs 0	1.19	0.004**
	3	27.64 ± 1.32	3 vs 0	1.12	0.111
	≥4	32.00 ± 0.00	≥4 vs 0	1.29	<0.001***
BMI	Underweight	21.33 ± 8.74			
	Normal weight	29.04 ± 0.84	Normal weight vs underweight	1.36	0.359
	Overweight	24.58 ± 1.31	Overweight vs underweight	1.15	0.675
	Obesity	29.89 ± 0.94	Obesity vs underweight	1.40	0.315
MMSE	No impairment	26.76 ± 1.13			
	Mild cognitive impairment	26.96 ± 1.29	Mild cognitive impairment vs no impairment	1.01	0.905
	Moderate dementia	29.00 ± 1.78	Moderate dementia vs no impairment	1.08	0.233
	Severe dementia	32.00 ± 0.00	Severe dementia vs no impairment	1.19	<0.001***
Barthel index	Independent	30.33 ± 1.67			
	Mild dependency	24.67 ± 1.86	Mild dependency vs independent	0.81	0.007**
	Moderate dependency	27.12 ± 1.42	Moderate dependency vs independent	0.89	0.100
	Severe dependency	26.63 ± 1.13	Severe dependency vs independent	0.88	0.033*
	Total dependency	29.67 ± 2.33	Total dependency vs independent	0.98	0.777
Hand grip	Normal strength	25.59 ± 1.79			
	Weak grip strength, risk of sarcopenia	27.62 ± 0.85	Weak grip strength vs normal strength	1.08	0.304
Tinetti index	Low fall risk	23.50 ± 4.16			
	Moderate fall risk	24.20 ± 4.10	Moderate fall risk vs low fall risk	1.03	0.896
	High fall risk	27.94 ± 0.61	High fall risk vs low fall risk	1.19	0.300


#### Geriatiric parameters correlated with caries

When correlating the number of decayed teeth with the geriatric assessment MMSE, it was found that if the MMSE increases by one unit, the number of decayed teeth decreases by 15%, which was statistically significant. If the Tinetti Index increases by one unit, the number of decayed teeth increases by 36% (supplementary Table A1).

#### Geriatiric parameters correlated with filled teeth 

When correlating the number of filled teeth with the patient’s gender, it was found that women had 58% fewer filled teeth compared to men, which was statistically significant. Married people had 76% more filled teeth compared to single people, while widowed people had 25% fewer filled teeth compared to single people. Patients living alone with help statistically significantly had 32% more filled teeth compared to patients living completely alone. If the Barthel index increases by one unit, the number of healthy teeth statistically significantly increases by 86% (supplementary Table S2).

#### Geriatiric parameters correlated with missing teeth 

When correlating the number of missing teeth with the patient’s gender, women had statistically significantly (37%) more missing teeth compared to men. Married people had 30% fewer missing teeth compared to single people, while widowed people had 3% fewer missing teeth compared to single people. Patients living alone with assistance had statistically significantly (24%) fewer missing teeth compared to patients living completely alone. Care-level 1 patients had 62% more missing teeth compared to care-level 0 patients, care-level 2 patients had 38% more missing teeth compared to care-level 0 patients, care-level 3 patients had 24% more missing teeth compared to care-level 0 patient, and care-level 4 patients had 46% fewer missing teeth compared to care-level 0 patients (Table 6).

**Table 6:** Poisson regression, variable, number of missing teeth

Correlated variable		Number of missing teeth (mean ± SD)	Comparison	Relative risk	p-value
Correlated variable		Number of missing teeth (mean ± SD)	Comparison	Relative risk	p-value
Sex	Male	15.76 ± 2.03			
	Female	21.35 ± 1.41	Female vs male	1.37	<0.001***
Marital status	Single	21.82 ± 2.76			
	Married	15.32 ± 1.89	Married vs single	0.70	0.817
	Widowed	21.10 ± 1.72	Widowed vs single	0.97	0.038*
Living environment	Alone	21.56 ± 1.44			
	Alone with help	15.64 ± 1.92	Alone with help vs alone	0.76	<0.001***
Level of care	0	16.52 ± 1.60			
	1	26.83 ± 1.89	1 vs 0	1.62	<0.001***
	2	22.21 ± 2.49	2 vs 0	1.38	0.020*
	3	20.45 ± 3.25	3 vs 0	1.24	0.233
	≥4	9.00 ± 0.00	≥4 vs 0	0.54	<0.001***
BMI	Underweight	15.33 ± 7.69			
	Normal weight	21.69 ± 1.76	Normal weight vs underweight	1.42	0.405
	Overweight	16.63 ± 1.86	Overweight vs underweight	1.08	0.849
	Obesity	23.11 ± 3.10	Obesity vs underweight	1.51	0.338
MMSE	No impairment	19.21 ± 1.77			
	Mild cognitive impairment	20.25 ± 1.82	Mild cognitive impairment vs no impairment	1.05	0.678
	Moderate dementia	22.00 ± 3.44	Moderate dementia vs no impairment	1.15	0.406
	Severe dementia	9.00 ± 0.00	Severe dementia vs no impairment	0.47	<0.001***
Barthel index	Independent	25.33 ± 20.02			
	Mild dependency	13.67 ± 1.67	Mild dependency vs independent	0.54	<0.001***
	Moderate dependency	20.23 ± 1.93	Moderate dependency vs independent	0.79	0.049*
	Severe dependency	18.96 ± 1.88	Severe dependency vs independent	0.75	0.013**
	Total dependency	20.67 ± 6.64	Total dependency vs independent	0.82	0.451
Hand grip	Normal strength	17.88 ± 2.25			
	Weak grip strength, risk of sarcopenia	20.29 ± 1.42	Weak grip strength vs normal strength	1.13	0.369
Tinetti index	Low fall risk	18.38 ± 4.21			
	Moderate fall risk	13.40 ± 4.89	Moderate fall risk vs low fall risk	0.73	0.419
	High fall risk	20.47 ± 1.26	High fall risk vs low fall risk	1.11	0.628
* p < 0.05; ** p < 0.01; *** p < 0.001.					

## DISCUSSION

Analysis of the data revealed a correlation between geriatric assessments, encompassing the physical, psychosocial, and functional abilities of elderly patients, and their dental health, as measured by the DMFT index and the numbers of healthy, missing, decayed, and filled teeth.

Particularly statistically significant associations were identified with care level, MMSE, Barthel Index, Tinetti Index, gender, marital status, and living environment. Higher care levels were clearly linked to a greater number of missing and decayed teeth, as well as a higher DMFT index. BMI showed that overweight individuals had more filled but fewer healthy teeth than underweight individuals. Cognitive impairment — especially severe dementia — was statistically significantly associated with a higher DMFT index and more missing teeth, while mild impairment was related only to the number of decayed teeth. Functional status, as assessed via the Barthel index, indicated that even mild dependence correlated with fewer filled and healthy teeth. Women demonstrated higher DMFT scores, more missing teeth, and fewer healthy and filled teeth than men. Marital status also influenced dental health, with married individuals having fewer missing teeth than their single counterparts.

In terms of care levels, most patients fell below care-level 4, resulting in limited data for this category. This suggests that the study population largely comprised relatively independent individuals.

No statistically significant relationship was found between grip strength and oral health in this study. This contrasts with other investigations suggesting that higher physical function, particularly handgrip strength, may be indicative of better oral health and related quality of life. A study involving 112 geriatric patients found such an association, and concluded: “Preventive physical rehabilitation practices, in addition to oral treatments, may be effective in improving oral health in the elderly”,^4^ while data from the Korean National Health and Nutrition Examination Survey with 10,607 final study participants also reported a statistically significant link between caries and grip strength.^15^ However, these findings often emphasize the need for further longitudinal research to explore causality. In our comparatively small cohort of 62 participants, the lack of statistical significance might be attributed to limited sample size, a relatively homogeneous level of functional ability, or minimal variance in grip strength among participants.

Similarly, although fall risk was not statistically significantly associated with oral health parameters in our data, indirect connections may exist, such as reduced mobility leading to decreased ability to attend dental appointments. Previous studies have suggested statistically significant associations between tooth loss and fall risk, proposing that oral health education, regular check-ups, and proper use of prostheses could help in fall prevention.^17^

It should be mentioned that, with 62 patients, the cohort of this study is relatively small. However, the quality of the population should be emphasized, which results from extensive general medical, geriatric, and dental patient data (as described in the Methods section) from highly motivated, compliant, and adherent patients. In addition, the descriptive statistics nevertheless show a strong diversity of patients in their general medical history as well as in their geriatric and dental findings. This speaks in favor of a broadly diversified and therefore high-quality cohort.

This study has some inherent limitations related to its design. The recruitment was conducted at a single center, which may somewhat limit the generalizability of the findings. Additionally, the exclusive inclusion of hospitalized patients and the lack of a control group may have introduced some selection bias. The higher proportion of female participants in the sample reflects the typical gender distribution in the geriatric population, supporting the representativeness in this context.

The cross-sectional design provides a valuable snapshot of the relationship between geriatric assessments and oral health status, measured by the DMFT index, although it does not allow conclusions regarding causality or temporal changes. Nevertheless, it offers important preliminary insights that can inform future longitudinal research.

While the cohort size of 62 patients is modest, it still provides meaningful data from a vulnerable and often underrepresented group. These findings contribute valuable knowledge to the field and lay the groundwork for larger-scale studies to further explore and confirm the observed associations. Future studies with a larger sample size would be needed to enable multivariable regression analyses, which could provide more robust and comprehensive results.

Overall, despite these design-related considerations, this study offers important contributions to understanding the oral health of hospitalized geriatric patients, highlighting relevant links between general health and oral status that can help guide future research and clinical practice.

This was not a prospective study with homogeneous groups but rather a reflection of the daily clinical setting, which could not be influenced. An age comparison between both groups reveals that women were, on average, only about one year older than men.

The questionnaire developed for this study consisted predominantly of binary (yes/no) and easily evaluable questions, which enabled the efficient collection of relevant dental information. More detailed items —such as oral hygiene habits and prosthetic use— were categorized and discussed during interviews, ensuring consistent data collection and interpretability.

The initial tooth status included the DMFT index, which serves as a valuable indicator of patients’ dental status. In our study, noticeably elevated DMFT scores were observed, suggesting poorer oral health conditions. No radiographs were taken due to the observational nature of the study and the fragile condition of the hospitalized patients, which may have led to some carious lesions remaining undetected and, consequently, to potentially even higher DMFT scores.

Another study with 352 geriatric patients similarly reported poor oral health in older people — particularly tooth loss and low number of functional occlusal units — highlighting the need to integrate dental care into general geriatric health concepts.^1^ High DMFT scores were largely attributable to missing teeth, alongside elevated plaque and root caries indices.^1^

A meta-analysis compiling global data on tooth loss and caries prevalence in individuals aged 45+ confirmed that untreated caries and edentulism are widespread, with considerable variation by age, country, and socioeconomic status.^2^ This underlines the global importance of targeted dental strategies for older adults.^2^

Previous research highlights the complexity of dental treatment in multimorbid, polymedicated older adults, as evidenced by findings from the DMS V survey.^19^ It advocates for a structured geriatric dental assessment, analogous to general geriatric assessments, including the evaluation of dental functional capacity through treatment ability, oral hygiene capability, and personal responsibility. That article concludes that dental care for older people must adapt to diverse life phases and challenges over time, calling for improved integration of dental services within the wider healthcare system, along with expanded training and education across disciplines.^19^

## CONCLUSION

This study demonstrates a correlation between the general medical history and geriatric assessments — i.e., the physical health, psychosocial and functional abilities of geriatric patients — and their dental health as measured by the DMFT index, number of healthy teeth, number of missing teeth, number of decayed teeth, and number of filled teeth. Particularly significant correlations were found in relation to level of care, MMSE, Barthel Index, Tinetti index, gender, marital status, and living environment.

A follow-up study is needed to investigate the correlation between specific geriatric diseases and dental health or disease. Dental findings can provide valuable insights into geriatric limitations and are an important component of the comprehensive geriatric assessment.

In conclusion, the results of the study underline the urgent need for specific dental treatment and care concepts for a holistic approach in geriatric patients.

## Appendix

**Table A1:** Poisson regression, variable, number of decayed teeth

Correlated variable		Number of decayed teeth (Mean ± SD)	Comparison	Relative risk	p-value
Correlated variable		Number of decayed teeth (mean ± SD)	Comparison	relative risk	p-value
Sex	Male	1.19 ± 0.62			
	Female	1.10 ± 0.51	Female vs male	0.90	0.880
Marital status	Single	1.36 ± 0.73			
	Married	1.42 ± 0.99	Married vs single	1.04	0.961
	Widowed	0.87 ± 0.43	Widowed vs single	0.64	0.526
Living environment	Alone	1.18 ± 0.39			
	Alone with help	1.05 ± 0.86	Alone with help vs alone	0.85	0.850
Level of care	0	1.21 ± 0.50			
	1	0.33 ± 0.33	1 vs 0	0.28	0.198
	2	0.79 ± 0.39	2 vs 0	0.61	0.434
	3	0.18 ± 0.18	3 vs 0	0.15	0.068
	≥4	19.00 ± 0.00	≥4 vs 0	15.74	<0.001***
BMI	Underweight	4.00 ± 4.00			
	Normal weight	1.46 ± 0.77	Normal weight vs underweight	0.37	0.298
	Overweight	0.50 ± 0.23	Overweight vs underweight	0.13	0.025*
	Obesity	0.78 ± 0.57	Obesity vs underweight	0.19	0.126
MMSE	No impairment	0.42 ± 0.17			
	Mild cognitive impairment	1.38 ± 0.61	Mild cognitive impairment vs no impairment	3.24	0.044*
	Moderate dementia	0.75 ± 0.48	Moderate dementia vs no impairment	1.77	0.400
	Severe dementia	19.00 ± 0.00	Severe dementia vs no impairment	44.79	<0.001***
Barthel index	Independent	0.67 ± 0.66			
	Mild dependency	2.00 ± 1.53	Mild dependency vs independent	3.00	0.285
	Moderate dependency	0.54 ± 0.24	Moderate dependency vs independent	0.81	0.817
	Severe dependency	1.00 ± 0.52	Severe dependency vs independent	1.49	0.673
	Total dependency	6.67 ± 6.17	Total dependency vs independent	10.00	0.039*
Hand grip	Normal strength	2.00 ± 0.81			
	Weak grip strength, risk of sarcopenia	0.78 ± 0.44	Weak grip strength vs normal strength	0.39	0.165
Tinetti index	Low fall risk	0.63 ± 0.62			
	Moderate fall risk	0.40 ± 0.40	Moderate fall risk vs low fall risk	0.64	0.730
	High fall risk	1.27 ± 0.48	High fall risk vs low fall risk	2.02	0.484
* p < 0.05; ** p < 0.01; *** p < 0.001.					

**Table A2:** Poisson regression, variable, number of filled teeth

Correlated variable		Number of filled teeth (Mean ± SD)	Comparison	relative risk	p-value
Correlated variable		Number of filled teeth (Mean ± SD)	Comparison	relative risk	p-value
Sex	Male	1.86 ± 0.46			
	Female	0.80 ± 0.29	Female vs male	0.42	0.045*
Marital status	Single	0.82 ± 0.38			
	Married	2.26 ± 0.62	Married vs single	2.76	0.048
	Widowed	0.61 ± 0.24	Widowed vs single	0.75	0.621
Living environment	Alone	0.77 ± 0.21			
	Alone with help	1.86 ± 0.58	Alone with help vs alone	2.32	0.038
Level of care	0	1.34 ± 0.34			
	1	0.33 ± 0.33	1 vs 0	0.25	0.140
	2	1.57 ± 0.72	2 vs 0	1.09	0.865
	3	0.73 ± 0.56	3 vs 0	0.54	0.425
	≥4	0.00 ± 0.00	≥4 vs 0	-	-
BMI	Underweight	0.33 ± 0.33			
	Normal weight	0.81 ± 0.36	Normal weight vs underweight	2.42	0.339
	Overweight	1.88 ± 0.46	Overweight vs underweight	5.62	0.042*
	Obesity	0.44 ± 0.44	Obesity vs underweight	1.33	0.818
MMSE	No impairment	1.09 ± 0.37			
	Mild cognitive impairment	1.42 ± 0.39	Mild cognitive impairment vs no impairment	1.29	0.542
	Moderate dementia	0.25 ± 0.25	Moderate dementia vs no impairment	0.23	0.113
	Severe dementia	0.00 ± 0.00	Severe dementia vs no impairment	–	–
Barthel index	Independent	0.67 ± 0.66			
	Mild dependency	3.33 ± 1.20	Mild dependency vs independent	4.99	0.064
	Moderate dependency	1.38 ± 0.47	Moderate dependency vs independent	2.08	0.407
	Severe dependency	0.81 ± 0.28	Severe dependency vs independent	1.22	0.821
	Total dependency	0.33 ± 0.33	Total dependency vs independent	0.50	0.548
Hand grip	Normal strength	1.41 ± 0.51			
	Weak grip strength, risk of sarcopenia	1.04 ± 0.29	Weak grip strength vs normal strength	0.74	0.496
Tinetti index	Low fall risk	1.13 ± 0.64			
	Moderate fall risk	2.00 ± 1.38	Moderate fall risk vs low fall risk	1.78	0.480
	High fall risk	1.06 ± 0.27	High fall risk vs low fall risk	0.94	0.921
* p < 0.05; ** p < 0.01; *** p < 0.001.					
